# Sensing dynamic human activity zones using geo-tagged big data in Greater London, UK during the COVID-19 pandemic

**DOI:** 10.1371/journal.pone.0277913

**Published:** 2023-01-20

**Authors:** Tongxin Chen, Di Zhu, Tao Cheng, Xiaowei Gao, Huanfa Chen

**Affiliations:** 1 SpaceTimeLab for Big Data Analytics, Department of Civil, Environmental and Geomatic Engineering, University College London, London, United Kingdom; 2 Department of Geography, Environment and Society, University of Minnesota, Twin Cities, MN, United States of America; 3 Centre for Advanced Spatial Analysis, Bartlett School of Architecture, University College London, London, United Kingdom; National Taiwan University, TAIWAN

## Abstract

Exploration of dynamic human activity gives significant insights into understanding the urban environment and can help to reinforce scientific urban management strategies. Lots of studies are arising regarding the significant human activity changes in global metropolises and regions affected by COVID-19 containment policies. However, the variations of human activity dynamics amid different phases divided by the non-pharmaceutical intervention policies (e.g., stay-at-home, lockdown) have not been investigated across urban areas in space and time and discussed with the urban characteristic determinants. In this study, we aim to explore the influence of different restriction phases on dynamic human activity through sensing human activity zones (HAZs) and their dominated urban characteristics. Herein, we proposed an explainable analysis framework to explore the HAZ variations consisting of three parts, i.e., footfall detection, HAZs delineation and the identification of relationships between urban characteristics and HAZs. In our study area of Greater London, United Kingdom, we first utilised the footfall detection method to extract human activity metrics (footfalls) counted by visits/stays at space and time from the anonymous mobile phone GPS trajectories. Then, we characterised HAZs based on the homogeneity of daily human footfalls at census output areas (OAs) during the predefined restriction phases in the UK. Lastly, we examined the feature importance of explanatory variables as the metric of the relationship between human activity and urban characteristics using machine learning classifiers. The results show that dynamic human activity exhibits statistically significant differences in terms of the HAZ distributions across restriction phases and is strongly associated with urban characteristics (e.g., specific land use types) during the COVID-19 pandemic. These findings can improve the understanding of the variation of human activity patterns during the pandemic and offer insights into city management resource allocation in urban areas concerning dynamic human activity.

## Introduction

City is a complex system reflecting human beings’ activities intertwined with natural environment [1–3]. Exploration of the dynamic human activity in urban areas can directly and comprehensively portray the social and economic activity units in space and time. As an indispensable population dynamic information in urban areas, it supports urban resource allocation in residence relocation, city planning, and public health emergency [4–8]. With the rapid development of ubiquitous location awareness technologies, massive amounts of geo-tagged big data (e.g., mobile phone GPS data, WiFi probe data and social media data) can be collected efficiently and continuously as real-time snapshots of individuals’ activity patterns. Then, such large volumes of human activity data regarding the spatio-temporal footprints tied with places and urban areas can provide the data-driven perspective to reveal the urban complex dynamics [9, 10].

The geo-tagged big data incorporating spatial and temporal information from citizen sensors provide a variety of approaches to characterising dynamic urban space with near-nature human activity patterns. In this regard, research focusing on sensing the urban zones with distinctive functions have utilised human activity to reveal the socioeconomic and urban geographical features [11, 12]. In parallel to the functional zones that urban space with specific functions constraining human being activities [11, 13, 14], human activity zone (HAZ) refers to a clustered area consisting of a combination of geospatial units exhibiting a certain similarity characterised by the human activity patterns [15]. As the representative of human activity dynamic in the urban areas, HAZ has been associated with amounts of identified urban functions to reveal the urban movements and structures [16–18], such as the intensity and evolution of urban space [19], the discrimination in the centre or sub-centre of urban areas [20] and identification and classification of function zones and land use areas [11, 12, 21, 22].

As COVID-19 and its variants continue to spread around the global cities, urban citizens’ lives have been changed significantly due to the pandemic containment policies (e.g., national lockdowns, stay-at-home orders) [23–25]. The widespread utilisation of geo-tagged big data from mobile phones has been involved in evaluating the tremendous human mobility shifting associated with social and public policies during the COVID-19 pandemic. Related works have addressed human mobility/activity pattern shiftings in cities, regions and countries by analysing aggregated mobility data sets (e.g., Google and Apple mobility data) [26–29]. In addition, the human activity shifting patterns captured by geo-tagged big data has been widely utilised for the evaluation of restriction policy effectiveness in contaminating COVID spreading [30–32], the socioeconomic impacts of the population mobility affected by restriction policies [33–36], and the social inequality in human mobility during the COVID-19 pandemic [37–41]. Previous studies exploring and interpreting human mobility pattern changes have concerned large geospatial districts in space and time. However, they neglect to disentangle the human activity variations in urban areas driven by different significant restriction policies and interpret such complex dynamics using urban characteristics during the pandemic.

Since citizens’ activity rhythms are heterogeneously distributed in urban places and areas, it is of great significance for investigating and characterising the human activity dynamic which can help to inform the re-opening measures of city management [38]. As many previous studies focus on the influence of restriction policies on the human activity dynamic at the very beginning periods since the pandemic outbreaks, they ignore the evaluation of the ongoing restriction or relaxation policies’ effects on the human activity restrictions. Considering different restriction policies imposed on the human activity dynamics across urban areas during the COVID-19 pandemic, the topic that HAZs variations and their relationship with urban characteristics need to be examined and discussed in detail. Accordingly, we focus on addressing the following questions: *How do HAZs change and evolve across urban areas driven by different restriction phases*? Further, *what are the main urban characteristics dominating the formulation of HAZs due to the different restriction phases*? To resolve these research questions, we propose an analysis framework to classify the variations of human activity patterns in urban geospatial areas approached by HAZs delineation, and identify the determinants by modelling the urban characteristics with HAZs in machine learning classifiers across the restriction phases.

In this study, we implemented our proposed analysis framework on the mobile phone GPS dataset during the eight spotlighted observation periods from Jan 1, 2020 to Feb 27, 2021 in Greater London, UK. We first utilised anonymous mobile phone GPS trajectory data to extract stays and aggregated them to footfalls at UK census output areas (OAs) as the representation of urban area units with human activity. Then, we portrayed the HAZs based on the homogeneity of human activity dynamic at the OA level for eight restriction phases, UK. At last, we examined the relationships between urban characteristics and human activity by identifying the feature importance in the machine learning classifiers. Our results demonstrate the delineation of significant HAZ variations in space and time, and the examination of relationships between generated HAZs and urban features impacted by the different pandemic restriction policies.

The remainder of this paper is organised as follows. The methods section introduces the research analysis framework and relevant human activity metrics in detail. The case study section presents the experiments implemented in our study area and the case study results. The discussion section reveals the implication and inspiration of empirical findings. Finally, the conclusions section concludes the contributions and shows the research limitations.

## Methods

### Analysis framework

**Fig 1:**
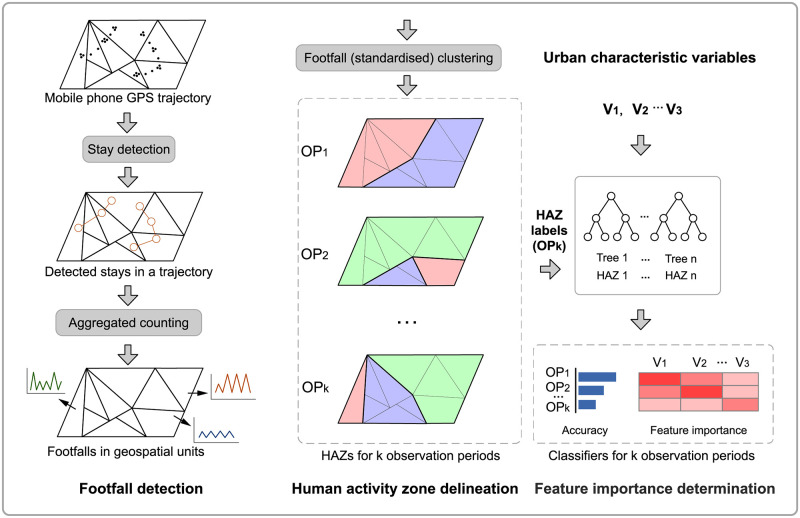
Analysis framework.

Fig 1 illustrates the three main processes in our analysis framework incorporating (1) footfall detection, (2) human activity zone (HAZ) delineation, and (3) Identifying relationships between static urban characteristics and dynamic HAZs. First, in the footfall detection part, a stay detection algorithm is used to retrieve stay points or stationary from a user’s position trajectory recorded as irregular GPS points. Next, footfall as a proxy of the human activity metric is calculated by aggregating detected stays coupled with geospatial unit information to enable us to evaluate the geospatial area with the human activity patterns. Second, in the process of delineating HAZ for the different observation periods, an agglomerative clustering algorithm is implemented to generate the HAZs, considering the homogeneity of temporal human activity patterns across various geospatial units. Lastly, identifying relationships between static urban characteristics and dynamic HAZs is approached by the feature importance determination in the machine learning classifiers.

### Footfall detection

As a metric of human activities in urban areas, footfall can be extracted from mobile phone GPS trajectory data [11, 42]. However, raw mobile phone trajectory data incorporating the sequential position records with temporal information cannot provide human activity semantic patterns (e.g., working, visiting or stay-at-home). Herein, the footfall detection process incorporates two steps: stay detection and aggregated counting.

Potential human activity can be described as a stay (i.e., stay points) that a single user spends some time in one place, i.e., the consecutive records of the user are at the same location during a time period [43–45]. Specifically, for one user’s position records, the sequence of GPS points *P* can be denoted as:

$$\begin{array}{c} P=p_{0}\overset{\Delta d_{0},\Delta t_{0}}{\to }p_{1}\overset{\Delta d_{1},\Delta t_{1}}{\to }\ldots p_{k}\overset{\Delta d_{k},\Delta t_{k}}{\to }\ldots p_{m-1}\overset{\Delta d_{m-1},\Delta t_{m-1}}{\to }p_{m},k=0,1,2\cdots m \end{array}$$ (1)

where the Δ*d*_*k*_ and Δ*t*_*k*_ denote the Euclidean distance and time intervals between two GPS points (*p*_*k*_ and *p*_*k* + 1_). Then, using the stay detection algorithm, stays set *S* can be detected from the sequence of GPS records *P*:

$$\begin{array}{c} S=\{s_{0},s_{1}\cdots s_{k}\cdots s_{n-1},s_{n}\},k=0,1,2\cdots n,n<m \end{array}$$ (2)

In this framework, the stay detection algorithm [46, 47] needs two preset parameters for input trajectory data, i.e., *d*_*max*_ (the maximum distance that records a user’s movement around from a point location to count as a stay) and *t*_*min*_ (the minimum duration time period that the records stay within time distance to qualify as a stay at the location). Hence, a stay can be detected while the Δ*d* is under *d*_*max*_ and the Δ*t* is above *t*_*min*_ between the first point and the last point of the GPS trajectory points.

Second, once the implementation of stay detection for each GPS trajectory of a single user in the data sets, we aggregate the counts of stays to footfalls as the proxy of human activity metric for a defined geospatial unit (e.g., census block, community) and temporal units (e.g., hourly, daily), respectively.

### Delineation of human activity zones for different observation periods

In relation to function zoning, a clustering method to retrieve urban function zones (a set of basic area clusters) with land use type information or specific social functions for citizens. We use HAZs to describe the clustering results that area clusters characterised by the similarity of footfalls (temporal human activity pattern) in the geospatial units. Briefly, the generation of HAZ is to extract *n* numbers of HAZs based on the footfall dynamic representing human activity temporal pattern from the *m* geospatial units (*n* < *m*).

A clustering strategy for achieving the HAZ generation across geospatial units and observation periods can be organised as follows. First, for a defined observation period *OP*_*k*_, suppose we obtain an aggregated footfall dataset in space and time, incorporating *m* geospatial units (e.g., grids, census blocks) with an observation period with *t* temporal units (e.g., hourly, daily, weekly). Then, a footfall matrix ***A*** with *m* rows and *t* columns can be organised from such spatio-temporal data sets. So, human activity (footfall volumes) ***A***_*i*, *j*_ at *i* th geospatial unit and *j* th temporal unit can be denoted as:

$$\begin{array}{c} A_{i,j},i=1,2,...,m,j=1,2,...,t \end{array}$$ (3)

Second, for the HAZ extraction considering the human activity pattern, we utilise a row-based standardisation process, i.e., performing the standardisation at the *i* th geospatial unit’s temporal footfall ***A***_*i*,:_. For example, as one geospatial area unit (*i*), we calculate the mean *μ*_*i*_ and variance *σ*_*i*_ from ***A***_*i*,:_ which can be denotes as:

$$\begin{array}{c} \mu_{i}=\frac{1}{t}\sum_{j=1}^{t}(A_{i,j}),\sigma_{i}=\sqrt{\frac{1}{t}\sum_{j=1}^{t}{\left(A_{i,j}-\mu_{i}\right)}^{2}},i=1,2,...,m,j=1,2,...,t \end{array}$$ (4)

After repeatedly standardised implementation at *m* geospatial units, we can get standardised footfall ***B***_*i*, *j*_ at the *i* th geospatial unit (row) and *j* temporal unit (column) which can be denoted as:

$$\begin{array}{c} B_{i,j}=\frac{A_{i,j}-\mu_{i}}{\sigma_{i}},i=1,2,...,m,j=1,2,...,t \end{array}$$ (5)

Here, the standardised footfall matrix ***B*** output from matrix ***A*** with *m* rows (geospatial units) and *t* columns (temporal units) is completed based on the row-based standardisation process.

Next, we utilise agglomerative clustering algorithm [48] at standardised footfall matrix ***B*** to retrieve *n* types of HAZs based on the human activity temporal pattern from *m* geospatial units. In this step, the distances across footfalls are represented by the classical Euclidean distance between time series vectors, and silhouette coefficient as a clustering optimisation metric (the highest/best value is 1 and the lowest/worst value is -1) [49] is used for the evaluation of pattern variation similarity across *m* geospatial areas’ footfall patterns at *t* temporal units.

Then, we maximise the silhouette coefficient to discriminate across clusters and determine the optimised number of clusters (HAZs) *n* (*n* < *m*). At last, we repeatedly implement the above procedures to generate the HAZs in *k* observation periods.

### Identifying the relationships between static urban characteristics and dynamic HAZs

To identify the relationships between static urban characteristics and dynamic HAZs in each observation period *OP*_*k*_, the static urban characteristics as explanatory variables are utilised to classify the dynamic human activity represented by HAZ labels (*n*) in the random forest (RF) classifier. First, we select the best RF classifier using the optimisation of accuracy as the model performance metric by the strategy of grid search and *k*-fold cross-validation. Second, the accuracy metric and feature importance indicator are outputted by this RF classifier. By executing previous steps on *k* time periods, we get *k* RF classifiers with accuracy representing a global relationship between urban features and HAZs, and feature importance representing a local relationship between each urban feature and HAZs, respectively.

The calculation of feature importance in the RF classifier is introduced as follows. By randomly selecting the subset of input variables, RF classifier as an ensemble of decision trees has received vast attention due to the reliable classification performance on high-dimension data and fast processing speed [50–52]. In this analysis, a general feature relevance indicator named *Gini importance* (*I*_*G*_) as a by-product from the RF classifier based on each urban characteristic variable (e.g., ***v***_1_ as an input feature vector in Fig 1) can be calculated from the inherent implementation of RF classifier.

In detail, each decision tree (e.g., Tree 1 of RF in Fig 1) as a basic classifier seeks an optimisation of splitting on a randomly selected subset of urban characteristic variables according to the *Gini impurity* as a splitting metric. While the decision trees are aggregated to the fitted RF classifier, the sum of *Gini impurity* criteria of feature variables in all splits is generally scaled to Gini importance [53–55]. The Gini importance *I*_*G*_ for an urban variable/feature ***v*** can be denoted as:

$$\begin{array}{c} I_{G}\left(v\right)=\sum_{T}\sum_{\tau }\Delta i_{v}\left(\tau ,T\right) \end{array}$$ (6)

where Δ**i**_***v***_(*τ*, *T*) is the decreased value of Gini impurity within the optimal split at node *τ* and tree *T*, respectively. Thus, *I*_*G*_(***v***) indicate the frequency/possibility of a feature *θ* is selected for splitting and the extent of discrimination in the HAZ labels (*n* types). So, the sum of all input variables *I*_*G*_ is equal to 1.

## Case study

### Data source and study area

#### The restriction phases during the COVID-19 pandemic in Greater London

With COVID-19 spreading in global cities, Greater London continues to undergo the diffusion of the viruses and variants as the metropolis with the highest number of confirmed cases in the UK. As an emergency response to the pandemic, the first national lockdown announced by the government started on Mar 23, 2020, following a series of restricted measures in the urban society, such as stay-at-home, and non-essential business closures. Our interest observation periods are eight policy restriction phases (422 days in total) discriminated by different national or local restriction laws [56] or policies [57] in Greater London from Jan 1, 2020 to Feb 27, 2021. In detail, Table 1 lists the key information of eight restriction phases.

**Table 1:** The eight policy restriction phases.

Restriction phases	Start date	End date	Days
Before lockdown	2020-01-01	2020-03-22	82
First national lockdown	2020-03-23	2020-07-03	103
Minimal lockdown restrictions	2020-07-04	2020-09-13	72
Reimposing restrictions	2020-09-14	2020-10-13	30
Three-tire restrictions	2020-10-14	2020-11-04	22
Second national lockdown	2020-11-05	2020-12-02	28
Four-tier restrictions	2020-12-03	2021-01-05	34
Third national lockdown	2021-01-06	2021-02-27	51

Then, this study focuses on Greater London at the output area (OA) level, i.e., Greater London’s 25,053 OAs that the geospatial areas the daily human activity patterns (footfalls) generate. The UK census output areas from small to large are ordered by UK postcode (PC), census output areas (OA), lower super output areas (LSOA), middle layer super output area (MSOA) and local authority (LA).

#### Mobile phone GPS trajectory data

**Fig 2:**
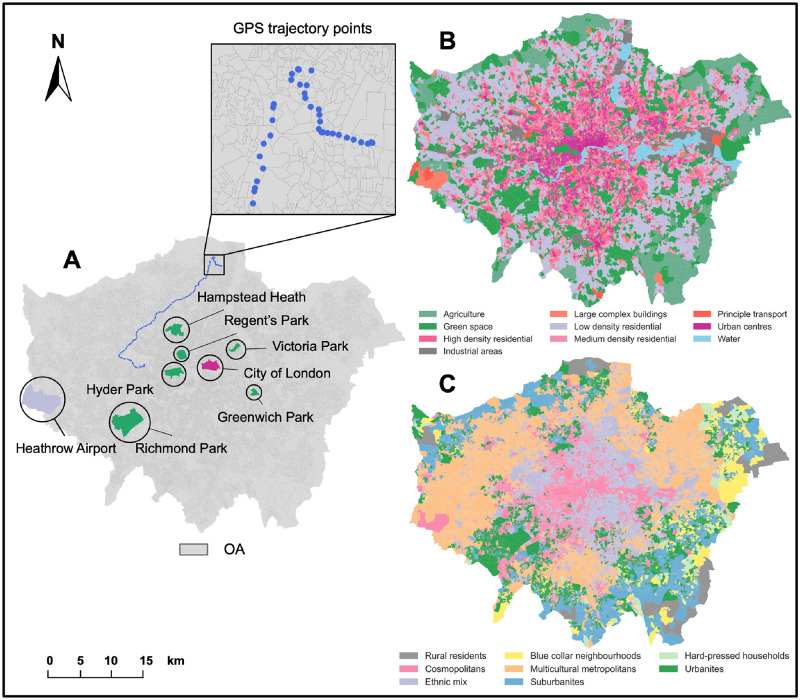
The mobile phone GPS trajectory sample and urban characteristic distribution at OA-level in Greater London. Map A shows a sample user’s GPS trajectory without the start and end points; Map B shows the distribution of OAs’ land use in ten types (we plot each OA in one land use represented by the maximum land use acreage in ten types). Map C shows the eight supergroups of OA socio-demographic classification. (Geographical boundary data source: Office for National Statistics licensed under the Open Government Licence v.3.0. Contains OS data © Crown copyright and database right 2022. Contains National Statistics data © Crown copyright and database right 2022.).

The human activity metric in terms of the footfalls (stays) is calculated from millions of anonymous users’ mobile phone GPS trajectory data provided by Location Sciences under GDPR compliance [58]. As the users are involved in broadly mobility-related apps (e.g., navigation, route planning, outdoor sports), this dataset is reliable for representing the human activity categories in the metropolis area. In general, there are 1153,637 users in Greater London as the main part (41.6%) of 2770,060 users in the whole UK data in our observation days (422 days). Considering the diverse applications of GPS data collection apps and a promised proportion in Greater London, our dataset can provide a good representation for exploring human activity patterns. Map A in Fig 2 shows a sample user’s GPS trajectory in our study without the starting and ending records as privacy protection. In addition, some interest areas’ boundaries are plotted as green parks, city centres and transportation facilities.

#### Urban characteristic data

In this study, we select the land use and socio-demographic data to represent urban static characteristics for analysis. First, land use data (Sep 2021 version) provided by Digimap [59] were downloaded from the ‘UKLand’ part in the ‘Verisk’ section. The dataset provides detailed land use information including the land use area with types across Greater London (map B in Fig 2). To clarify, the land use types are aggregated into ten main types for describing the London land use breakdowns: high-density residential with retail and commercial sites, urban centres—mainly commercial/retail with residential pockets, medium density residential with high streets and amenities, low-density residential with amenities (suburbs and small villages/hamlets), large complex buildings various use (travel/ recreation/ retail), principle transport, green space and recreational land, industrial areas, agriculture, water. Considering the OAs as the unit of analysis in this study, we calculate each type of land use area for every OA in Greater London so that each OA can be characterised as 10 different land use acreage (*km*^2^).

Second, the latest London OAs socio-demographic classification data are provided by Office for National Statistics [60]. It depicts the grouped characteristics of socio-demographic variables at OAs (2011 census) and obtains three-level classifications (i.e., 8 super groups, 24 groups and 67 subgroups). In this study, we select eight super groups for analysis, including rural residents, cosmopolitans, ethnic mix, blue collar neighbourhoods, multicultural metropolitan, suburbanites, hard-pressed households, and urbanites. The OA classification map (eight super groups) is shown as map C in Fig 2.

### Human activity changes in Greater London during restriction phases

To enable the footfall as a proxy of human activity at each OA in Greater London, we define the stay (stationary) as a user spending at least 5 mins within a distance of 50 meters spatial radius from a given GPS trajectory. Specifically, these two parameters are consistent with the previous stay detection work [61], which allows us to find some users’ significant visiting behaviours at places. Next, the detected stays are aggregated to the footfalls at OA and daily levels in space and time, respectively. Then, the generation of HAZs is employed on the spatio-temporal matrix with daily human footfalls on each OA in London (25,053 OAs * 422 days in total).

**Fig 3:**
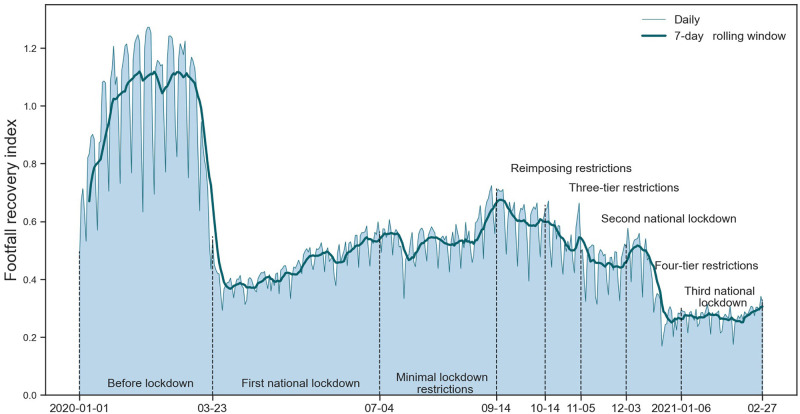
Daily footfall recovery index in Greater London from 2020-01-01 to 2021-02-07. Daily and 7-day rolling window observations are shown as light blue and dark blue lines, respectively. The eight policy restriction phases are distinctively separated by vertical lines labelled with the start/end date.

In order to assess the human activity recovery in Greater London, we calculate the recovery index of footfalls by comparing it with the benchmark of the daily average footfall volume from Jan 1, 2020 to Feb 29, 2020 (60 days). From a global point of view, Fig 3 depicts the daily footfall recovery index by comparing the daily footfall volumes (from Jan 1, 2020 to Feb 27, 2021) with the benchmark of whole Greater London areas (i.e., the accumulation of footfall volumes of all OAs). In general, the footfall recovery index of Greater London obtains various levels during the eight policy restriction periods. Specifically, we observe the footfall level of Greater London experienced a tremendous reduction of about 65% after the first national lockdown (Mar 23, 2020) with a series of restricted measures, such as closures of non-essential business, entertainment, public infrastructures and stay-at-home order.

In addition, a minor increase is observed at the end of the minimal lockdown restrictions, followed by a distinct decline at the start of reimposing restrictions (2020-09-14) with a ‘rule of six’ coming into force. Next, another similar footfall change with a sharp decline is found during the four-tier restrictions that overlapped with the Christmas holidays. At last, the overall daily trend of Greater London during all restriction phases has not returned to the normal level (i.e., 100%) since the first national lockdown (Mar 23, 2020) imposed on the whole country.

### Dynamic HAZ delineations in Greater London during the COVID-19 pandemic

To generally understand the influences of restriction policies on the spatial distribution and corresponding temporal footfall dynamics of HAZs during the pandemic, we delineate the HAZs during all observation periods and the eight restriction phases, respectively. The first part describes the distribution of HAZs and their footfall pattern during all observation periods. The second part examines the dynamic HAZs and related footfall patterns affected by eight distinctive restriction phases.

**Fig 4:**
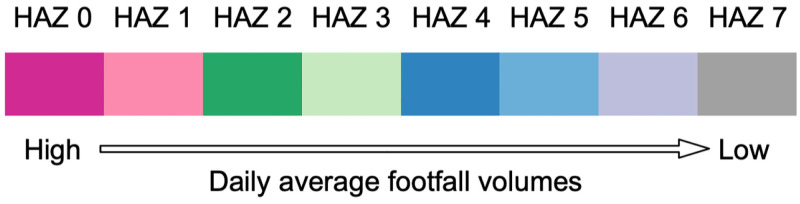
The colour palette used in HAZ delineation maps.

For visualisation consistency in HAZ maps, the HAZ indices in each map are ranked by the daily average footfall volumes of HAZs from high-level to low-level and following a constant colour palette shown in Fig 4. Under such defined rules, different coloured HAZs indicate the relatively ‘busier areas’ and ‘less busy areas’ in terms of footfall volumes. Additionally, each HAZ footfall temporal pattern is represented by the mean of the standardised footfall pattern of all corresponding clustered OAs. In addition, the footfall temporal pattern of each HAZ is plotted as the same colour as the correspondent HAZ under the maps.

#### HAZs during all observation periods from 2020-01-01 to 2021-02-27

**Fig 5:**
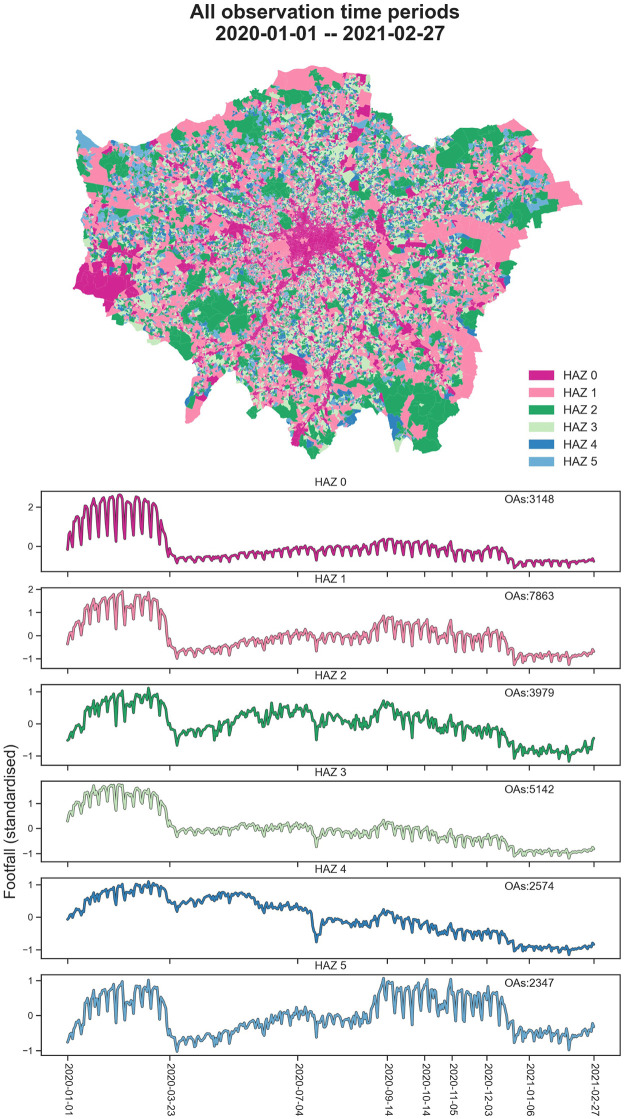
HAZs (top) and corresponding footfall patterns (bottom) of Greater London during all observation periods. The HAZs are ranked by the daily average footfall volumes from high level to low level. The temporal footfall variations for each HAZ are represented by the mean of all related OAs’ footfalls. (Geographical boundary data source: Office for National Statistics licensed under the Open Government Licence v.3.0. Contains OS data © Crown copyright and database right 2022. Contains National Statistics data © Crown copyright and database right 2022.).

To delineate the HAZs during all observation periods from 2020-01-01 to 2021-02-27, we performed the proposed function zoning (footfall clustering) based on the footfall patterns at OAs to generate HAZs in Greater London. First, we get the optimised cluster numbers (6) by maximising the silhouette coefficient. The OAs with homogeneous standardised footfall patterns are labelled as the same cluster and grouped as a HAZ in the agglomerative clustering step. The HAZs and corresponding clustered footfall patterns of Greater London during all periods are depicted in Fig 5. Here, the HAZs (from HAZ 0 to HAZ 5) are ordered by the daily average footfall of HAZ, i.e., 71.6, 35.7, 22.8, 20.3, 17.4 and 15.1, respectively. Then, the temporal dynamic of each HAZ is characterised by the mean of all OAs’ footfalls.

To illustrate, the spatial distributions of HAZs of Greater London during all observation periods generally match the distinctive urban structures in terms of human activity discrimination. For example, as the busiest areas, the urban areas in HAZ 0 (red) are mainly narrowed in the city centre (the areas around City of London), the clustered areas on the west of London (Heathrow Airport area) and linear shape areas diffusing from urban centre to urban suburb (the main road network of London). In the temporal examination, all footfall volumes of HAZs obtained reductions sharply after the announced first national lockdown (2020-02-23), but HAZ 4 is observed with a relatively slight decline affected by the policy compared to other HAZs.

Additionally, unlike the majority of HAZs (HAZ 0, HAZ 1, HAZ 3 and HAZ 4) with ongoing low-level footfall volumes after the first national lockdown (2020-03-23), HAZ 2 (green) and HAZ 5 (light blue) are observed that human activity ‘recovered’ during some observation periods (e.g., from 2020-09-14 to 2020-10-14). In detail, human activity recovery in HAZ 2 is found at the end of the first national lockdown phase (July 4, 2020) and the end of the minimal lockdown restrictions phase (Sep 14, 2020). Several urban green spaces are involved in HAZ 2, e.g., Richmond Park, Regent’s Park, Hampstead Heath and Victoria Park, highlighted in map A of Fig 2. On the contrary, the recovery of human activity in HAZ 5 as the low-level footfall volume areas started on Sep 14, 2020 and lasted until the middle of the four-tier restrictions.

#### Dynamic HAZs influenced by the eight different restriction phases

**Fig 6:**
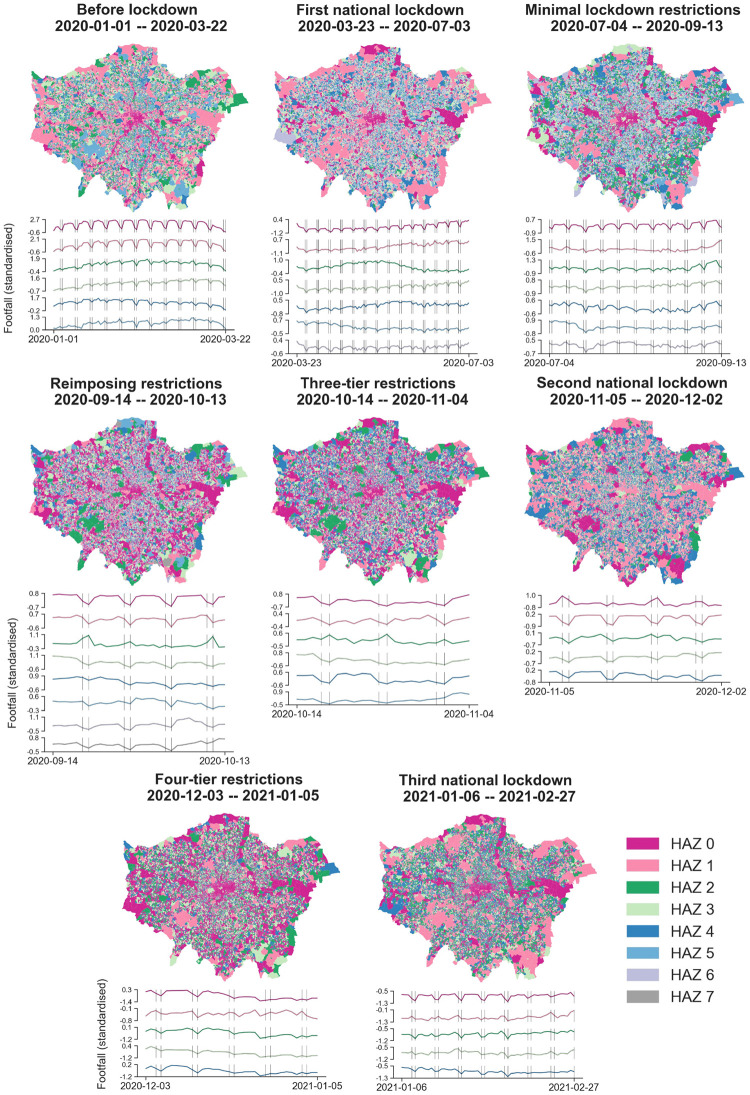
HAZs of Greater London in eight distinctive policy restriction phases. For each phase, the HAZs are extracted from footfall patterns, and the HAZ numbers are 6, 7, 7, 8, 6, 5, 5, 5, respectively. The vertical lines in the temporal footfall figures denote weekends. (Geographical boundary data source: Office for National Statistics licensed under the Open Government Licence v.3.0. Contains OS data © Crown copyright and database right 2022. Contains National Statistics data © Crown copyright and database right 2022.).

To portray the variations of HAZs in response to the eight different restriction phase, we get the optimised HAZ numbers (i.e., 6, 7, 7, 8, 6, 5, 5, 5) for the before lockdown phase, the first national lockdown phase, the minimal lockdown restrictions phase, the reimposing restrictions phase, the three-tire restrictions phase, the second national lockdown phase, the four-tier restrictions phase and the third national lockdown phase, respectively. The HAZs and corresponding clustered human activity patterns of Greater London during eight policy restriction periods are denoted in Fig 6. And Table 2 shows the daily average footfall volumes and OA numbers of HAZs in each restriction phase. Like Fig 5, we use the same palette rule shown as Fig 4 to describe HAZs and their footfall patterns.

As we can observe in Fig 6, the impact of COVID-19 response restriction measures significantly and heterogeneously affect human activities across urban areas in Greater London. Overall, human activity patterns at different restriction periods are substantially discriminated in spatial distribution. We can distinctively observe all HAZs obtaining informative heterogeneous regionalisation in the Greater London map. In the sub-figure of the before lockdown phase, we can find six types of HAZs distributed in Greater London. The distribution of HAZ 0 (red) is quite similar to the HAZ 0 in Fig 5 that the busiest urban areas are distinctly narrowed by linear shape from the city centre to the fringe, while the other HAZs have not been observed following this pattern in the morphology. Though the human activity volume levels are different across HAZs, substantial weekly trends in temporal footfall patterns can be found in HAZ 0 and HAZ 1 on busy weekdays.

Then, the dynamic HAZ classifications imply a complex set of typologies in terms of the changes in human activity patterns across the pandemic restrictions phases. In the first lockdown phase, we observe the distribution of HAZs has been reconstructed with the busiest areas changing to a non-consecutive shape compared to the HAZs map of the before lockdown phase. Regarding the footfall patterns of HAZs, the busy-weekday areas (HAZ 0 plotted as red) still obtain the highest footfall volume levels and concentrate on the urban centres. Then, HAZ 1 (pinks) is observed that a slightly increasing trend started at the middle stage of this phase (the end of May 2020). The related government’s amendments to the regulations and new rules have effects from May 31, 2020 are that allowing people to meet outside in groups of up to six and phased re-opening of schools [62].

In the next three restriction phases, we find a sustained similarity of human activity patterns in space between the reimposing restrictions and the three-tier restrictions. Though globally the human activity volumes are observed declines in the two time period denoted by Fig 3, the distributions of HAZs in the two maps resemble each other in terms of our classification. In the second national lockdown phase, it is obvious that the hottest areas (HAZ 0) have not concentrated on the city centre but are dispersed in several areas with a busy-weekend pattern in the metropolis. Next, in the four-tier restrictions phase, we observe a steady decrease in the footfall level in HAZ 0 with a busy-weekday pattern, but a slight increase in the footfall level in HAZ 1 with a busy-weekend trend during the Christmas holidays. At last, we observe that HAZ 0 and HAZ 1 as the top two busy areas, obtain distinctly converse weekly patterns in the third national lockdown phase. In particular, the busiest areas (each HAZ 0) stay clustered in the city centre both before and during the pandemic with a significant weekly pattern in footfalls. Here, though the city centre has not portrayed in the busiest areas (HAZ 0) in the second national lockdown phase, it remains the second-busiest area as a part of HAZ 1, and obtaining a similar footfall pattern with former phases (i.e., busy-weekday trend).

**Table 2:** The Daily average footfall volumes and OA numbers of HAZs in eight policy restriction phases.

Restriction phases	HAZ 0	HAZ 1	HAZ 2	HAZ 3	HAZ 4	HAZ 5	HAZ 6	HAZ 7
**Before lockdown**	174.0(1872)	62.1(8064)	47.1(4303)	34.6(4214)	27.2(4079)	26.6(2539)	–	–
**First national lockdown**	57.2(2136)	28.1(4956)	22.1(1272)	21.4(5621)	19.0(4756)	18.9(2677)	18.4(3635)	–
**Minimal lockdown restrictions**	61.5(3440)	34.7(1631)	32.7(3929)	26.5(4848)	20.3(3996)	20.0(3001)	18.9(4208)	–
**Reimposing restrictions**	46.0(7565)	43.4(3452)	39.9(889)	23.8(4594)	22.5(2709)	19.0(2953)	18.5(1442)	17.7(1449)
**Three-tire restrictions**	41.8(7120)	26.7(3937)	24.9(1692)	22.9(4793)	21.2(5386)	19.9(2125)	–	–
**Second national lockdown**	39.6(817)	32.8(8959)	18.8(2539)	18.8(4222)	16.9(8516)	–	–	–
**Four-tier restrictions**	30.2(8622)	16.8(2192)	16.3(5744)	14.9(5228)	12.3(3267)	–	–	–
**Third national lockdown**	24.4(4520)	22.3(4082)	11.0(7513)	10.2(3204)	10.1(5734)	–	–	–

**Fig 7:**
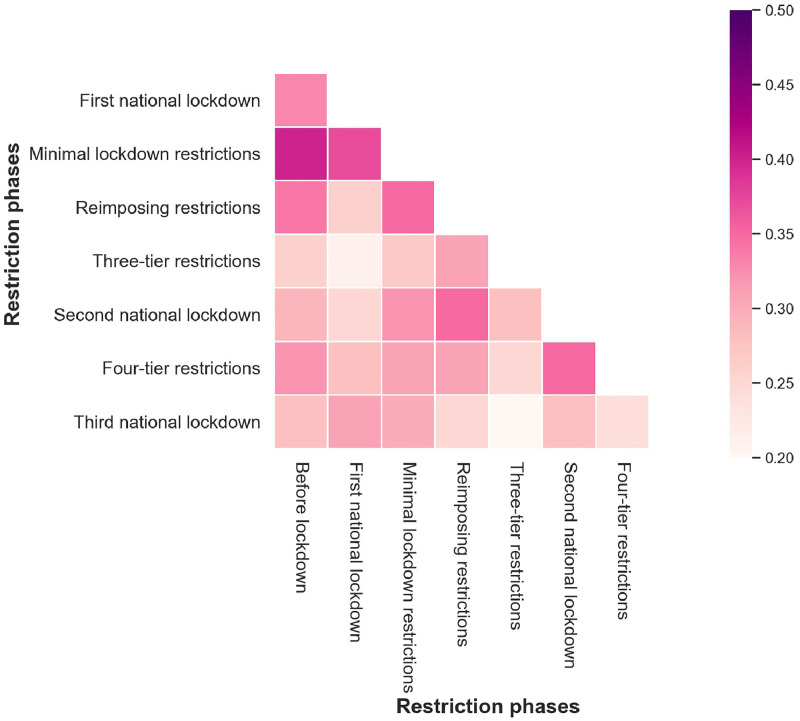
The difference between the HAZs from eight restriction phases.

As the dynamic HAZs amid the different restriction phases, we tested the difference between HAZ types in eight restriction phases using Pearson’s Contingency Coefficient and the results are shown in Fig 7. Significantly, the HAZ classification in the before lockdown phase and the first national lockdown obtain high associations with the HAZs during the minimal lockdown restrictions.

### The relationships between static urban characteristics and the discrimination of HAZs during the pandemic

**Fig 8:**
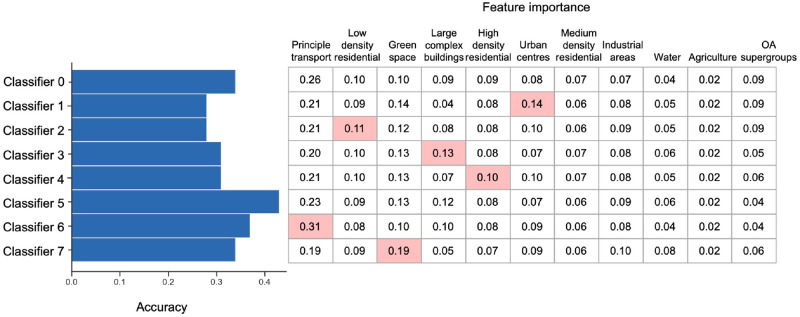
The accuracy values (left) and feature importance values of urban characteristics (right) in RF classifiers of different restriction phases. There are ‘Before lockdown’ (Classifier 0), ‘First national lockdown’ (Classifier 1), ‘Minimal lockdown restrictions’ (Classifier 2), ‘Reimposing restrictions’ (Classifier 3), ‘Three-tire restrictions’ (Classifier 4), ‘Second national lockdown’ (Classifier 5), ‘Four-tier restrictions’ (Classifier 6), and ‘Third national lockdown’ (Classifier 7).

To assess the relationships between static urban characteristics and human activity represented by HAZs in different restriction phases, we trained the RF classifiers based on HAZ labels and selected urban features (OA supergroups and land use areas) as input variables. To be specific, the input urban characteristic matrix (***X***) consist of 25,053 rows (OA numbers) and 11 columns (OA supergroups categories and 10 types of land use acreage values). In the hyper-parameter procedure, the grid search and *k*-fold cross-validation (*k* was 10 and with 15 iterations) are used to select the optimised RF classifier with outputting accuracy and feature importance for each restriction phase. Then, the results of multi-classification accuracy values and corresponding feature importance values of RF classifiers at eight restriction phases are shown in Fig 8.

Globally, the accuracy values (left) of RF classifiers have not shown the promised performances in discriminating dynamic HAZs as all the values are below 0.5. Additionally, the prediction accuracy of the second national lockdown model (classifier 5) reaches the highest at 0.44 and the minimal lockdown restrictions model reaches the lowest at 0.27 (classifier 2), respectively. Locally, the right part denotes the feature importance of each RF classifier from every observation period. We observe several relatively high feature importance values of the urban features from different observation periods. The highest value (above 0.1) of each urban characteristic in different observation phases is highlighted as pink cells. Significantly, it denotes that the principle transports variable contributes to the significant effects on the discrimination in HAZs from the classifiers, and the importance value reaches the highest level at the four-tier restrictions (Classifier 6). In addition, the feature importance of green space in the third national lockdown (Classifier 7) is the highest value compared to other classifiers of restriction phases.

## Discussion

This study has aimed to investigate the variations of human activities across urban areas using geo-tagged big data in Greater London during the COVID-19 pandemic. Our analytic framework has demonstrated significant changes in human activity patterns represented by the HAZs in space and time. Following our proposed analysis framework, footfalls as the human activity metrics can be aggregated on stays detected from raw mobile GPS data leveraged by the stop detection algorithm, and HAZs can be efficiently generated based upon the OAs’ footfalls using the agglomerative clustering algorithm. Then, our classification of HAZs in urban geospatial areas can be an effective way of exploring the relationship between human activity patterns and urban characteristic variables from the RF classifiers. The results facilitate our understanding of how different containment policies influence human activity patterns in space and time across urban areas.

**Fig 9:**
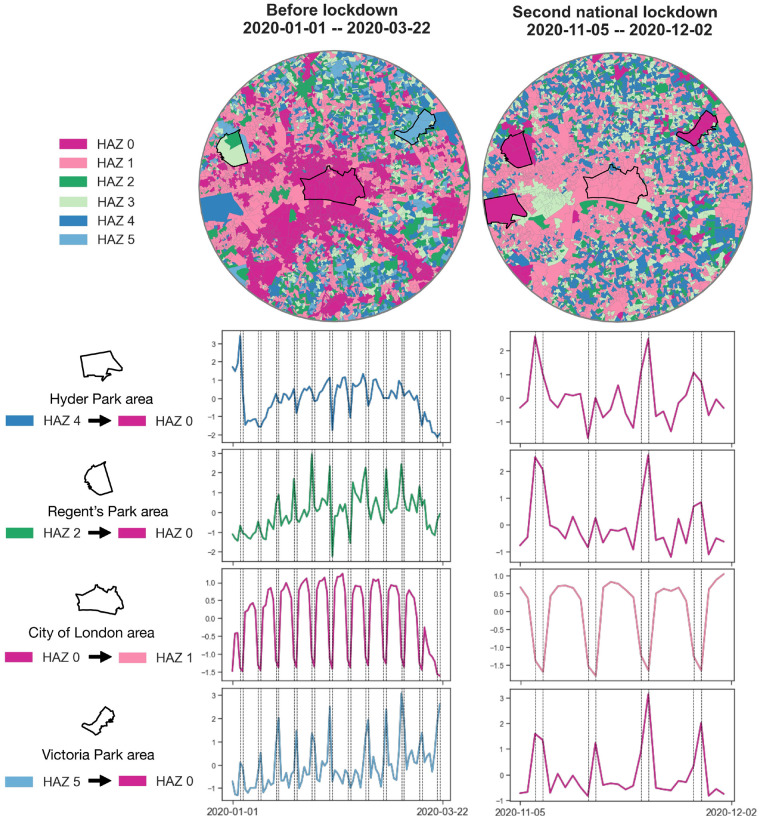
The HAZs within the urban centre’s buffer area (6 km) in the before lockdown phase and the second national lockdown phase. Geographical boundary data source: Office for National Statistics licensed under the Open Government Licence v.3.0. Contains OS data © Crown copyright and database right 2022. Contains National Statistics data © Crown copyright and database right 2022.

Our findings have demonstrated that human activity changes in urban areas obtain a roughly general decrease or increase in terms of footfall volumes but also the heterogeneous spatial patterns of HAZs affected by the different restriction policies. Inherently, the variations in human activity patterns represented by HAZs are associated with specific land use types across the urban areas in Greater London. In terms of the urban centre examination, we plot several places and their footfall patterns (standardised) in the before lockdown and second national lockdown phases within the urban centre areas in Fig 9. The busiest areas around the City of London in the before lockdown phase had shifted to the surrounding parks in the second national lockdown phase. Obviously, such busy area displacements are strongly connected to the restriction policies, i.e., the closure of non-essential high street businesses, and citizens can meet one person from outside their ‘support bubble’ outdoors rather than inside the home in the second national lockdown phase.

Though the COVID-19 pandemic has caused spatial displacements in HAZs across urban areas, the results also show that the connections between human activity and land use remain stable in terms of the footfall temporal patterns rather than footfall volumes influenced by the restriction policies. For example, the human activities in the urban centre areas in the first or second national lockdown phase have tremendously decreased compared to the normal phase, the footfall temporal pattern of these areas remains the busy-weekday trend as the workplace function effect contributing to the classification of HAZs (e.g., the two footfall patterns of City of London, Victoria Park or Regent’s Park area shown in Fig 9). An alternative explanation is that dynamic populations still obtain high requirements for visiting or working across the urban leisure/workplace areas during the pandemic, so the sensed human activity dynamics with similar commuting behaviour patterns are captured as a classification in terms of the footfall patterns. Besides, the human activity patterns in relation to land use functionality at some specific type of place can be affected by restriction policies. For example, the busy-weekday footfall patterns in the Hyder Park area during the before lockdown phase are found to change to a busy-weekend trend in the second national lockdown phase. It is highlighted that workplace-related human behaviours (e.g., communing) in these places have reduced during the second national lockdown.

Considering other urban characteristics, the principle of transport and green space with a higher level of feature importance than other urban features during different restriction phases denote these land use types dominate the HAZ formulation affected by the restriction policies. In other words, the influences in the human activity of these land use obtain a higher level than others and such variations have been captured by the HAZs discrimination. On the contrary, the weak effect of socio-demographic features (OA classification data) and other land use types on the discrimination in the HAZs denotes that static urban characteristics cannot explain the dynamic human activity either before the pandemic or during the pandemic.

Disaggregating some of the results presented here could identify the types of HAZs that are significantly associated with human behaviours shifting in relation to urban function variations during the pandemic crisis. Considering the human activity patterns in urban areas affected by restriction policies not only can help to strengthen the policy evaluation but also might provide evidence for further developing tailored recommendations in several city management topics. For instance, city management resources (e.g., policing patrolling) might be more efficiently used if considering some specific types of areas obtaining the distinctively human activity changes, while our previous work has proved the strong connections between HAZ and crime change during the pandemic [15]. Additionally, further public health-related social measures in restriction or relaxation in the city (e.g., mobility restriction, work-from-home suggestions) can be allocated to specific urban areas while evaluating the dynamic urban areas associated with human activities.

## Conclusions

In conclusion, this research analysed human activity variations in space and time in small urban areas and explored the associations between human activity and static urban characteristics in Greater London during several pandemic restriction phases from 2020-01-01 to 2021-02-27. The results enhance our understanding of how human activity patterns could be influenced by different policies and affect the discriminant spatio-temporal patterns across urban areas. The exploration of spatio-temporal variations of human activity intertwined with urban land use can be adopted as an approach to disentangle some of the urban complexity.

The findings strengthen our knowledge concerning dynamic human activities in urban areas amid different restriction phases and give insight into that the spatial-temporal changes of human activity are related (obviously not limited) to urban characteristic variables. So, pubic-related strategies could be developed considering the combination of human activity-related variables and urban features.

One limitation of this study is that footfall as a proxy of human activity cannot reflect the information on travelling across the urban areas, which cannot portray human activity and further discuss other inequality of characteristics during the pandemic in a comprehensive way. In this initial exploration, it has not been possible to examine human activity patterns considering an hourly reflection of a city’s daily phenomena (e.g., commuting, traffic peaks). Additionally, a combination of human activity and other interesting urban variables needs to be generally considered in future research.
